# In an Age of Open Access to Research Policies: Physician and Public Health NGO Staff Research Use and Policy Awareness

**DOI:** 10.1371/journal.pone.0129708

**Published:** 2015-07-22

**Authors:** Laura L. Moorhead, Cheryl Holzmeyer, Lauren A. Maggio, Ryan M. Steinberg, John Willinsky

**Affiliations:** 1 Graduate School of Education, Stanford University, Stanford, California, United States of America; 2 Lane Medical Library, Stanford University School of Medicine, Stanford, California, United States of America; Katholieke Universiteit Leuven, BELGIUM

## Abstract

**Introduction:**

Through funding agency and publisher policies, an increasing proportion of the health sciences literature is being made open access. Such an increase in access raises questions about the awareness and potential utilization of this literature by those working in health fields.

**Methods:**

A sample of physicians (*N*=336) and public health non-governmental organization (NGO) staff (*N*=92) were provided with relatively complete access to the research literature indexed in PubMed, as well as access to the point-of-care service UpToDate, for up to one year, with their usage monitored through the tracking of web-log data. The physicians also participated in a one-month trial of relatively complete or limited access.

**Results:**

The study found that participants' research interests were not satisfied by article abstracts alone nor, in the case of the physicians, by a clinical summary service such as UpToDate. On average, a third of the physicians viewed research a little more frequently than once a week, while two-thirds of the public health NGO staff viewed more than three articles a week. Those articles were published since the 2008 adoption of the NIH Public Access Policy, as well as prior to 2008 and during the maximum 12-month embargo period. A portion of the articles in each period was already open access, but complete access encouraged a viewing of more research articles.

**Conclusion:**

Those working in health fields will utilize more research in the course of their work as a result of (a) increasing open access to research, (b) improving awareness of and preparation for this access, and (c) adjusting public and open access policies to maximize the extent of potential access, through reduction in embargo periods and access to pre-policy literature.

## Introduction

In 2008, the National Institutes of Health (NIH) adopted a Public Access Policy that requires recipients of NIH research funding to make all resulting peer-reviewed journal articles freely available online within a year of publication. The NIH Public Access Policy is part of a global trend toward public or open access in scholarly publishing. This trend involves not only new open access policies adopted by research funding agencies, including the NIH, Canadian Institutes of Health Research, Wellcome Trust, and Gates Foundation. It also comprises open access publishers, such as Public Library of Science (PLOS) and BioMed Central; journals providing full or partial open access, including *Journal of the American Medical Association* (*JAMA*), *New England Journal of Medicine* (*NEJM*), and *Environmental Health Perspectives* (*EHP*); and academic publishers offering open access titles, from Springer and Elsevier to Oxford University Press [1]. While still representing only a portion of the research literature, the number of freely available research articles is growing annually, with one-fifth of the literature published in 2009 available as open access [2].

As a policy leader and major funder of health research, the NIH has, through its Public Access Policy, placed health literature at the forefront of the move toward open access to research. As well, the NIH allows researchers to budget as needed for the Article Processing Charges that apply to many open access journals in the medical and public health fields. To assess the potential public implications of this growing access, this study examines the extent to which two groups working in the field of health—physicians and public health non-profit, non-governmental organization (NGO) staff—use this literature as part of their professional practice when they have free access to it. Provided with relatively complete online access to research literature indexed in PubMed for up to a year through a study web portal, participants’ research access was tracked, including the publication status of articles accessed, in terms of two dimensions of the NIH policy’s implementation timeframe: 1) the NIH policy’s initial 12-month embargo period (during which those subject to the policy are not required to provide public access for up to 12 months after publication) and 2) the period prior to the policy’s adoption and implementation. Though specific timeframes differ, variable constraints on research article access across publication dates—from the most recent to older articles—are common to many open access policies. The study also considers participants’ prior awareness of the NIH Public Access Policy, if any.

The NIH policy is intended “to advance science and improve human health” [3], with the assumption being that “public access” will enable health professionals as well as the general public, to draw on this body of research in ways that will improve health. As Dr. Elias Zerhouni, former director of the NIH, explained, the goal of the NIH Public Access Policy is to generate “change in the landscape of how scientific information is made available to the public” [4]. What is known is that research abstracts are becoming more popular with diverse publics [5]. Physicians, in particular, rely on abstracts to guide their clinical decision making [6]. Given the availability of abstracts, this study asks whether there is also public interest and value in having access to complete research articles. For despite shifting academic publishing norms toward open access, assessing the value of open access initiatives, such as the NIH’s policy, both in terms of their impact on the number of articles publicly available and their precedents in the realm of public and open access policy, remains difficult [7].

This study contributes to a much needed body of knowledge, given that a “dearth of research” remains on the use and value of public access to peer-reviewed research articles [8]. Researchers have noted that studies of physicians’ use of online research behaviors are rare, particularly in clinical settings and over an extended period of time [9], [10]. With regard to public health NGOs, there are recent studies examining community-based organizations’ research capacity and practices [11], though not focused specifically on the use of and need for research articles. For policy-makers and others, the question remains “whether free access to the scientific literature is making a difference in non-research contexts, such as teaching, medical practice, industry, and government policy-making” [8], as well as in research contexts outside of universities. There is an opportunity for additional analysis of both the public value and the need for research access, particularly to guide open access policy-making and implementation for diverse public stakeholders.

## Methods

The study utilizes 1) quantitative analysis of web-log data on all participants’ usage of the study web portal to access research resources; and 2) qualitative data from surveys and/or interviews regarding research practices, tailored to the study’s two principal participant groups (physicians and public health NGO staff). In this paper, we report on the quantitative analysis of web-log data and its overarching policy implications, as well as participants’ policy awareness. We will examine qualitative material on participants’ research uses and practices in future papers, with attention to the particularities of the participant groups studied and to broader issues raised by participant discussions of research challenges and priorities. The Stanford University Institutional Review Board approved this study and participants consented to participate through the study’s portal (described below).

### Participant Recruitment

The study examines the use of research by two participant groups, who provide an opportunity to assess open access policies on a wide spectrum of health practice, ranging from patient care to public health advocacy and policy change. The study sought two groups for whom research is a potentially relevant tool in carrying out their professional mandates to promote health, including preventative medicine and the advancement of health equity.

From March 2013 through June 2013, we enrolled subjects in the study’s two arms: 1) physicians practicing in the United States (U.S.) and 2) public health non-profit NGO staff in the U.S. Recruitment materials advertised free access to the Stanford University Library’s online research resources. Physician participants were told that the study would provide them with 11 months of free access to the Library’s entire collection of biomedical research and one month of limited access. NGO participants were informed that they would receive 12 months of complete access. All participants were informed that their access to this research literature would be recorded throughout the study.

#### Physicians

We recruited 336 physicians via professional organizations, list-servs, and social media (Facebook, LinkedIn and Twitter). Physicians could enroll immediately at any time using a weblink included in all communications. Upon registration, we verified the physicians’ licensure through the U.S. Centers for Medicare & Medicaid Services’ National Plan and Provider Enumeration System. Among these participants, 185 (55.1%) reported having had prior access to a point-of-care research summary subscription service, such as UpToDate and DynaMed. As well, 29 (8.6%) had email addresses ending in.edu, indicating an educational affiliation with potential access to research articles through an academic institution, although this did not prove to be statistically significant factor in participant’s research article views.

#### Public Health NGO Staff

We recruited 92 public health participants from 46 organizations by emailing U.S.-based non-profit NGOs addressing an array of public health issues, from environmental health and homelessness to primary care and patient advocacy. Recruitment sought to include organizations working at a range of levels, from those focused on local communities to those oriented toward municipal, regional, state, national, and, occasionally, international scales. Hence study participants came from both community-based organizations (CBOs) and national research and advocacy organizations. Of the 65 public health NGO staff that completed online surveys, 47 (72.3%) reported having previously used PubMed in their work.

### Web Portal and Article Access Measures

Participants registered for the study via a custom web portal, created by Stanford software developers (S1 Screenshot). The portal offered participants two research resources: PubMed (a life sciences and health research database) and UpToDate (a clinical decision-support service). The portal acted as a gateway, through PubMed or UpToDate, to the Stanford University Library online collection of approximately 9,000 health journal subscriptions. We selected PubMed based on its prevalence in clinical care [12], [13] and, more generally, public health. The inclusion of UpToDate was directed toward physicians and acted as a constant in the treatment (complete research access) and control (partial research access) conditions in the first and twelfth months to assess physician information use.

A proxy server recorded participants’ portal sessions in a standard web-log format, and a relational database housed the log data. All session data, including timestamps, unique session strings, search terms, requested resources (identified by PMIDs), referring pages, and IP addresses, were recorded. Data collection began whenever a participant clicked into the portal. The record includes clicks to view research abstracts, research articles, and UpToDate entries. Abstracts and research articles could be accessed through PubMed or UpToDate. An article’s PMID number enabled identification of year of publication, publication type, journal title, article title, and PubMed Central (open access) status. Multiple clicks by a participant on the same resource within a 30-minute session counted that resource view only once. Articles that were already open access, independent of our study enabling Stanford Library access, were determined by checking for their inclusion in PubMed Central (PMC), PubMed’s free full-text subset, within 24 hours of participant article access.

### Data Collection

#### Physicians

To establish whether complete access affected research use and how such use related to the use of UpToDate by physicians, a trial design was introduced by randomly assigning the physicians (*N* = 336) to two groups. In the first month, MD1 (*n* = 168) had complete library access; MD2 (*n* = 168) acted as a c*ontrol*, with library access limited to UpToDate. While MD2 participants could click to view articles, they only saw those that were currently open access (roughly one in five articles; Björk et al., 2010). This arrangement was reversed for MD1 and MD2 in month 12 (with all physicians having complete access in months two to ten). Participants were not initially informed of their group, though the consent form detailed the study design. Participants in both groups were notified after their first month as to which group they had been assigned and that everyone at that point had full access to journal articles available through Stanford University Library. Upon completion of the online component of the study, we conducted semi-structured phone interviews, approximately 30 to 60 minutes in length, with 38 physicians about their priorities and use of journal articles in clinical, research and other professional practices, as well as their awareness of the NIH Public Access Policy and prior PubMed use.

#### Public Health NGO Staff

Each of the 46 non-profit organizations identified one participant to take part in an initial online survey and semi-structured interview discussing research practices, for a total of 46 initial surveys and interviews. We requested that organizations identify the staff member most responsible for research to take part in this portion of the study. We also offered an optional orientation to PubMed following the interviews, for those expressing interest via the survey; 34 participants expressed interest. Some organizations identified additional staff interested in the research access and these staff were also registered for the web portal. For the duration of the 12-month study, all 92 participants enrolled through the study web portal were given complete access to the journal collection of Stanford University Library available via PubMed and UpToDate. Finally, at the end of the year-long study, online exit surveys were conducted examining participants’ evaluations of the study’s research access.

## Results

This paper focuses on quantitative analysis of research use via the web-log data and its overarching policy implications, with qualitative materials to be examined in future papers. The results of this analysis are organized into the following four sections: 1) Participants’ awareness of the NIH Public Access Policy; 2) Extent of participants’ research article access during study; 3) Publication dates and access rights of research articles viewed; and 4) Physicians research article and UpToDate access during treatment and control periods. When interpreting the data, it is crucial to keep in mind the differences in sample size and fields of practice of the participant groups studied. For that reason, the results for MDs and for NGO staff are reported separately.

### 1) Participants’ Awareness of the NIH Public Access Policy

This study assumes that greater public awareness of this new stream of research access, and similar open access policies, is likely to correlate with greater research investigation and use that is important to professional practice in an array of contexts.

Though this study began more than six years after the adoption of the NIH Policy in April 2008, the majority of all participants had never heard of the policy and its potential relevance in meeting their research needs (Table 1).

**Table 1:** MD (*n* = 38) and Public Health NGO staff (*n* = 65) response to question: “Before participating in this study, had you heard of the NIH Public Access Policy?”

	MD *n* (%)	NGO Staff *n* (%)
Yes	6 (15.8)	15 (23.1)
Maybe	3 (7.9)	7 (10.8)
No	29 (76.3)	43 (66.1)

### 2) Extent of Participants’ Research Article Access During Study

Although 336 physicians signed up at the study’s web portal to participate, only 115 (34.2%) viewed one or more research articles through the portal over the course of the 11 months of full access to the literature, while 145 (43.2%) used the portal without viewing any research articles, and 76 (22.6%) did not use the portal after registering (Table 2). Physicians gave a range of reasons for low or non-use, including lack of time, having research access outside the study, forgetting that they had access, forgetting how to access the site, and not having the portal embedded in their daily workflow (i.e., inaccessible through shared work machines).

**Table 2:** Use of portal and viewing of research articles among MDs (*N* = 336) and public health NGO staff (*N* = 92) for 11 and 12 months, respectively.

	MD *n* (%)	NGO Staff *n* (%)
Made no use of portal	76 (22.6)	16 (17.4)
Used portal without viewing articles	145 (43.2)	14 (15.2)
Viewed one or more research articles	115 (34.2)	62 (67.4)
Total	336 (100)	92 (100)

**Fig 1:**
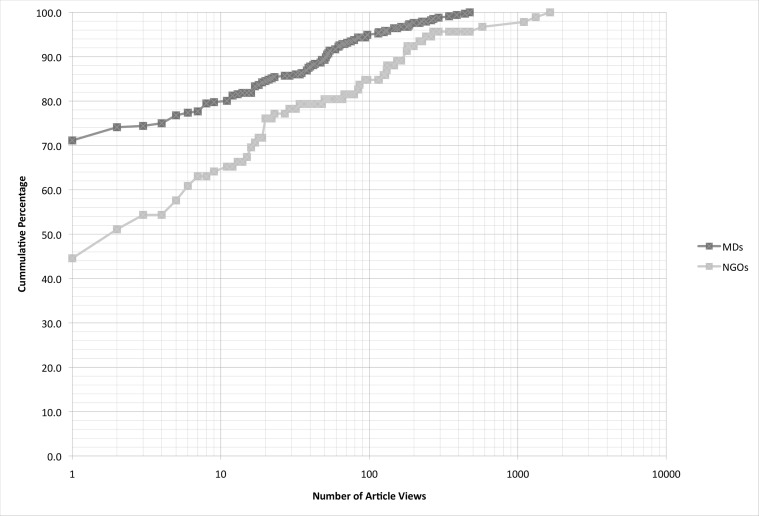
Cumulative percentage of article views for physicians (*N* = 336) over 11 months and public health NGO staff (n = 92) over 12 months.

The cumulative percentage of article views by participant illustrates the relative distribution of light to heavy users among physicians with 11 months of access and public health NGO staff with 12 months of access (Fig 1).

Among the public health NGO staff, the majority or 62 (67.4%) viewed one or more research articles during their 12 months of access, while 16 (17.4%) never accessed the site. Among the reasons for non-use of the portal, three NGO staff were associated with an organization that dissolved shortly after their registration to the web portal. Other reasons for low or non-use include parallels with reasons given by physicians, as well as changed work plans.

The 115 physicians who viewed one or more research article, viewed 5,984 articles in total, with a mean of 52 articles per physician over 11 months, working out to 1.2 articles per week on average (Table 3). The 62 NGO participants who viewed one or more article, viewed 7,201 articles total, with a mean of 116 per participant, which worked out to 2.2 articles per week on average. In terms of relative demand for full-text articles, physicians looked at 3.6 abstracts (freely available in PubMed) for every article viewed, while NGO staff accessed 3.7 abstracts for each article viewed, on average.

**Table 3:** Among MDs (*n* = 115) and public health NGO staff (*n* = 62) who viewed one or more research articles, mean use and weekly views-per-participant for 11 and 12 months, respectively.

	MD	NGO Staff
Total articles viewed	5,984	7,201
Mean articles viewed per participant (*SD*)	52.0 (87.3)	116.1 (302.0
Mean articles viewed weekly per participant (*SD*)	1.2 (2.0)	2.2 (5.8)

**Fig 2:**
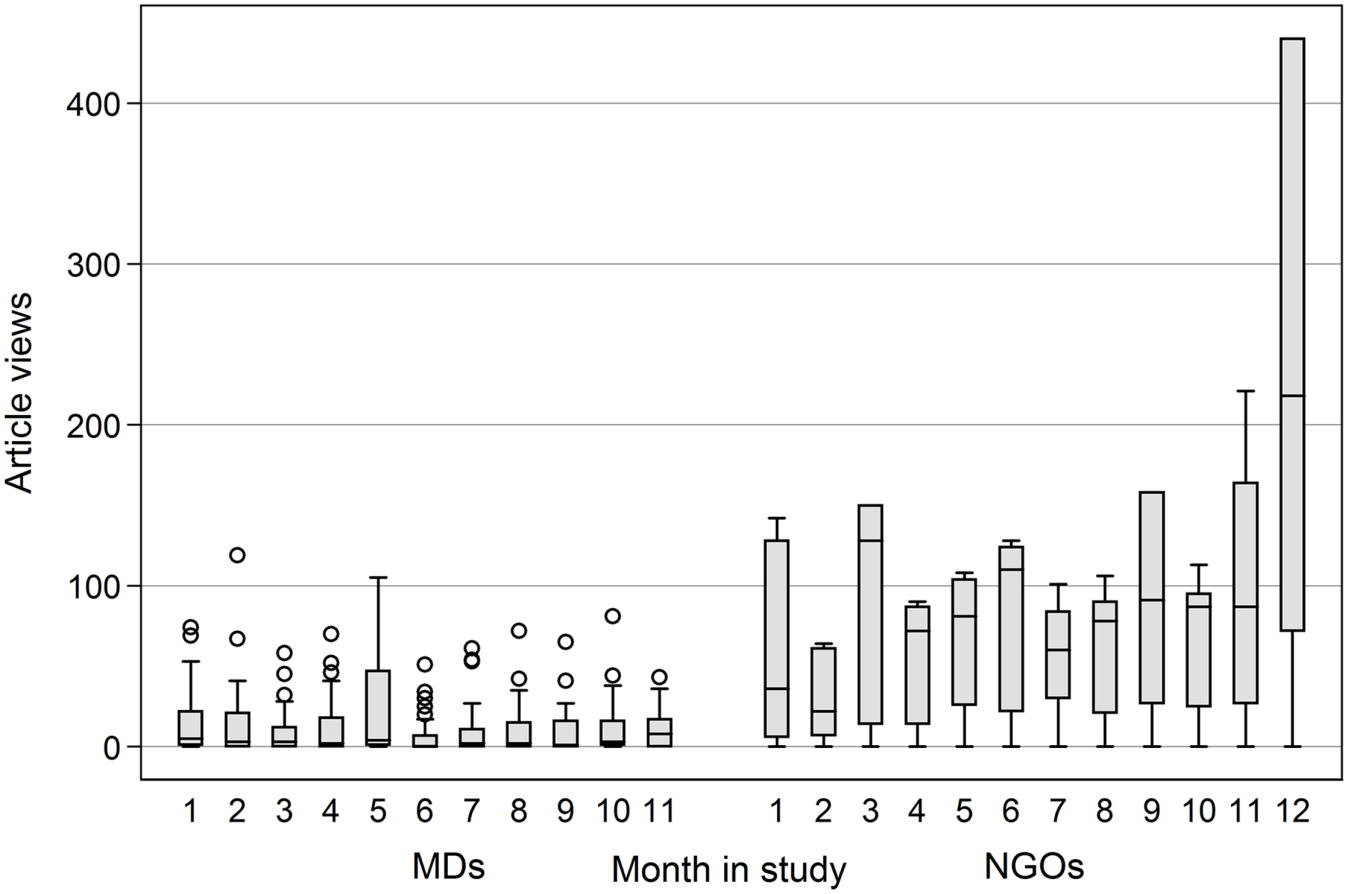
Total article views by month for MDs (*n* = 115) and public health NGO staff (*n* = 62) over 11 months and 12 months, respectively.

Over the course of the study, the monthly total of article views varied up and down (Fig 2).

While the physicians viewed fewer articles on average and cumulatively than the NGO staff, physician use was marked by outlying heavy users in every month but the 5th and 11th (as indicated by the dots in Fig 2). The NGO staff had a more even distribution of use, though with higher standard deviations, and with considerable activity in their twelfth and final month in the study.

**Fig 3:**
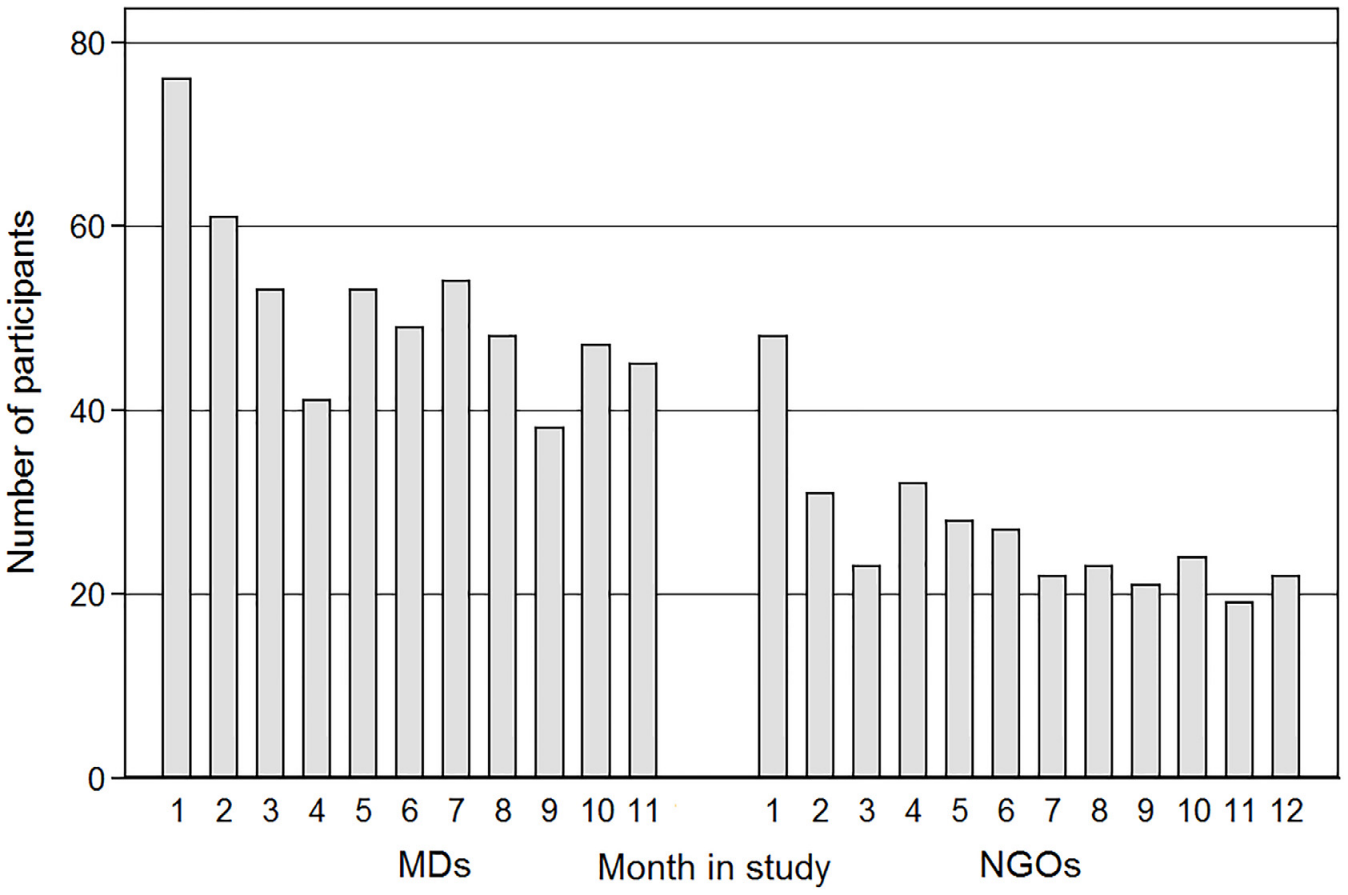
Monthly totals of the MDs (*n* = 115) and public health NGO staff (*n* = 62) viewing at least one research article, over 11 months and 12 months, respectively.

The number of participants viewing articles in each month declined from the initial months in the study among both physicians and NGO staff (Fig 3), while the article use by those who continued to access articles increased, given the relative stability of average article use (Fig 2). Among the physicians (*n* = 115; 55.7%) and public health NGO staff (*n* = 62; 58.1%), who viewed at least one article, the majority saw less than 20 articles over the course of the study. Approximately 10% (*n* = 17) of the physicians and 18% (*n* = 14) of public health NGO staff viewed over 100 articles. Among the journals with the most article views, the physicians (Table 4) and public health NGO (Table 5) had only three titles in common, *JAMA*, *NEJM*, and *Lancet*.

**Table 4:** Top ten journals by article views for MDs (*n* = 115) over 11 months.

Publication	MD Article Views
*The New England Journal of Medicine*	221
*JAMA*: *The Journal of the American Medical Association*	114
*International Journal of Radiation Oncology*, *Biology*, *Physics*	90
*Obstetrics and Gynecology*	67
*Annals of Emergency Medicine*	66
*BMJ*	62
*Annals of Internal Medicine*	59
*Lancet*	56
*Circulation*	45
*Journal of the American Academy of Dermatology*	43

**Table 5:** Top ten journals by article views for NGOs (*n* = 62) over 12 months.

Publication	NGO Article Views
*The American Journal of Clinical Nutrition*	323
*JAMA*: *the Journal of the American Medical Association*	190
*The Journal of Nutrition*	144
*American Journal of Public Health*	120
*The New England Journal of Medicine*	104
*BMJ*	98
*Diabetes Care*	81
*Journal of Public Health Management and Practice*	68
*Lancet*	68
*PLOS ONE*	68

### 3) Publication Dates and Access Rights of Research Articles Viewed

The NIH Public Access Policy includes, as many open access policies do, an embargo period after publication, of 12 months, during which a publisher is not required to make the article freely available. Close to half of the articles viewed by the physicians (49.9%) and somewhat less by the public health NGO staff (42.4%) were viewed within 12 months of being published (Table 6). In addition, 21.7% of the articles viewed by physicians and 24.0% of those viewed by the public health NGO staff were published prior to the adoption of the NIH policy in 2008.

**Table 6:** Articles viewed by publication date for MDs (*n* = 115) over 11 months and for public health NGO staff (*n* = 62) over 12 months.

Date of Publication	MD Totals *n* (%)	NGO Totals *n* (%)
Embargoed (within 12m of viewing)	2,983 (49.9)	3,050 (42.4)
From 2008 to within 12m of viewing	1,701 (28.4)	2,423 (33.6)
Pre-policy (prior to 2008)	1,300 (21.7)	1,728 (24.0)
Total	5,984 (100)	7,201 (100)

Nonetheless, a number of the articles viewed within 12 months of publication were already open access, whether from publishers depositing articles in PMC early or due to being published in open access journals. Here, 20.8% of the articles viewed in this 12-month period by physicians and 32.2% of those viewed by public health NGO staff were open access (Table 7). Similar proportions of the articles viewed from prior to the policy’s adoption were open access, with 22.3% for physicians and 38.6% for public health NGO staff. While as noted, a number of access policies from funding agencies and journals affect the availability of PubMed content, 14.2% (1,928) of the articles viewed by our participants were identified as having received NIH funding.

**Table 7:** Open Access articles viewed by publication date for MDs (*n* = 115) over 11 months and for public health NGO staff (*n* = 62) over 12 months.

		MDs			NGOs	
Date of Publication	Open Access *n* (%)	Non-OA *n* (%)	Totals *n* (%)	Open Access *n* (%)	Non-OA *n* (%)	Totals *n* (%)
Embargoed (within 12m of viewing)	620 (20.8)	2,363 (79.2)	2,983 (100)	982 (32.2)	2,068 (67.8)	3,050 (100)
From 2008 to within 12m of viewing	437 (25.7)	1,264 (74.3)	1,701 (100)	1,150 (47.5)	1,273 (52.5)	2,423 (100)
Pre-policy (prior to 2008)	290 (22.3)	1,010 (77.7)	1,300 (100)	667 (38.6)	1,061 (61.4)	1,728 (100)
Total	1,347 (22.5)	4,637 (77.5)	5,984 (100)	2,799 (39.9)	4,402 (61.1)	7,201 (100)

Among the participants in this study, the public health NGO staff viewed a higher proportion of open access articles (38.9%), compared with physicians (22.59%) (Chi-square test *p*<0.001). The two most active public health NGO organizations in article views subsequently reported, when queried by email, that they had no greater awareness of their higher use of open access articles nor had they sought out open access articles specifically.

### 4) Physicians’ Research Article and UpToDate Access During Treatment and Control Periods

In assessing whether full access to the research literature was of value to physicians with UpToDate, we found that when physicians had complete access (MD1 in month 1 and MD2 in month 12), those who looked at articles viewed significantly more abstracts and articles (using a paired *t*-test) than when they only had partial access (MD1 in month 12 and MD2 in month 1), which was limited to articles that were already open access (Table 7). However, complete and partial access conditions did not significantly affect physician use of UpToDate, which was equally available under both conditions (Table 8).

**Table 8:** Effect of complete research access and partial research access (limited to open access articles) for physicians’ UpToDate views, abstracts views, and clicks to view articles, during month one and 12.

	Complete Access Mean (*SD*)	Partial Access Mean (*SD*)	Value of *t*
UpToDate entry views (*n* = 38)	10.3 (3.8)	7.1 (3.7)	1.91
Abstract views (*n* = 37)	11.7 (3.8)	7.0 (3.4)	2.02*
Clicks to view articles (*n* = 29)^a^	9.8 (3.5)	4.7 (3.0)	3.53**

^a^ Under partial access, clicks to view articles resulted in either an open access article or a paywall, while under complete access it led to the article.

* *t* significant at *p* < .05.

** *t* significant at *p* < .01.

Neither gender nor having had prior access to DynaMed or UpToDate significantly affected physicians’ viewing of research articles over the course of the study. However, on average, physicians who graduated prior to 1990 accessed approximately 32 articles (*SD* = 7.5) over the 11 months, which is significantly more (*p* = 0.027) than those who graduated after 1991 and who accessed approximately 12 articles (*SD* = 5.7) over the same period.

## Discussion

### 1) Participants’ Awareness of the NIH Public Access Policy

Given that the majority of both physicians (76%) and public health NGO staff (66.2%) prior to participating in the study had not heard of the NIH Public Access Policy or were uncertain about its mandates, there is a clear need and opportunity for the NIH and other federal agencies to better publicize and explain public access policies. This seems especially true given that this study took place approximately six years after the NIH policy’s adoption. The Public Access Policy opens up to the public some 90,000 new health articles each year [3], and it is likely that this research is underutilized.

More effective and targeted publicity of the NIH Public Access Policy would help make the most of the policy in its current form, a theme addressed in the policy literature [14]. It would also help to better utilize other investments in health and research, by making research available to a wider array of practitioners and leveraging the contributions of multiple sectors to promoting public health, through broader access to peer-reviewed research for all. This emphasis on wide-ranging, cross-sector communication to better leverage resources dovetails with calls to “break down silos” in professional practice [15, 16], as well as within and between government agencies, including public health agencies [17]. Such silos and limited communication result in missed opportunities for cross-sectoral collaboration, innovation, and preventative health policies.

### 2) Extent of Participants’ Research Article Access During Study

Our findings attest to how, for a portion of the participants, their research interests were not satisfied by article abstracts alone nor, in the case of the physicians, by, in addition, a clinical summary service such as UpToDate. On average, a third of the physicians viewed research a little more frequently than once a week, with those who graduated prior to 1990 viewing significantly more articles than those with a more recent graduation date, while two-thirds of the public health NGO staff viewed more than three articles a week. While a click to view an article does not necessarily mean that an article was read, it does provide evidence of interest in full-text article access among a sizeable portion of each of these two groups, interest that extended across the year of access. The results of the trial in months one and 12 for the physicians also suggest that complete access leads to more viewing of research than current partial access and that this additional viewing of the research does not take away from the time given to consulting UpToDate but is supplementing that service. In addition, NGO participants’ spike in research use in the last month may be due to “warehousing” of articles for future use. Indeed, some participants mentioned that they intended to compile article libraries on topics relevant to their organization’s work, suggesting the longer-term value of full-text research access to organizational development.

In assessing web-log data, it is also important to consider the diverse tasks and wide variation in work cycles that may shape research use frequencies. Research access for potentially infrequent tasks—such as developing or updating patient or public policy guidelines, preparing testimony for public hearings, or grant writing—may nonetheless matter greatly to the quality and outcomes of the work. Sporadic use, from the standpoint of third-party evaluations of web-log data, does not necessarily mean insignificant use.

### 3) Publication Dates and Access Rights of Research Articles Viewed

The participants in this study demonstrated the potential importance of public access to a wide range of research, in terms of publication date. An unfortunate shortcoming of the current NIH Public Access Policy is that it neglects to address public access to articles that were published prior to the policy. Between a fifth and a quarter of the articles viewed were published prior to the policy’s adoption in 2008. Access to older biomedical and public health articles can enable publics to read and evaluate “classic articles” [18], including those capturing research landmarks such as “the establishment of a link between cholesterol and arteriosclerosis” and “a diagnostic test for color blindness” [18]. Study participants accessed articles back to 1878. These articles may be important to understanding particular health conditions, medical history, or health policy history, among other dimensions of interest. Post-2008 articles also tend to contain pre-2008 citations, whether “classic articles” or more mundane studies. Fully assessing the arguments and understanding the contexts of these recent articles necessitates broader research access to their sources. Such access may be especially important for engaging with certain types of research, such as systematic reviews and studies of long-term research trends [19, 20].

Similarly, this study has relevance for assessing the potential implications of initial embargo periods in policies such as the NIH Public Access Policy. Nearly half of all articles viewed by participants would be subject to the policy’s maximum embargo of 12 months, if the research were funded by the NIH. Our results show a reduction (4.9% for MDs and 15.3% for NGOs) in the proportion of open access articles viewed in the initial 12-month period (compared to the period from 2008 to within 12 months of viewing). This suggests that embargos are having an effect at this point on what is publicly available. While the NIH states that 12 months is the *maximum* embargo, some publishers allow for reduced periods, while others make 12 months their policy: “All Elsevier proprietary journals, where the author has identified themselves as being NIH funded, will submit the accepted author manuscript on behalf of the author, to PMC to be made publicly available 12 months after final publication” [21].

The policy implications of these findings on how the embargo period relates to physician and public health NGO staff use of the literature go beyond the NIH Public Access Policy. The 12-month embargo period was also included in the U.S. government’s most recent policy statement to all federal agencies on “Increasing Access to the Results of Federally Funded Scientific Research,” in a 2013 memorandum issued by the Office of Science and Technology Policy. Significantly, this recent policy statement includes a stipulation that an agency may make exceptions to the maximum 12-month embargo period, to “*tailor its plan as necessary to address* the objectives articulated in this memorandum, as well as *the challenges and public interests that are unique to each field and mission combination*” (p. 3; emphasis added) [22]. In addition, agencies “shall also provide a mechanism for stakeholders to petition for changing the embargo period for a specific field by presenting evidence demonstrating that the plan would be inconsistent with the objectives articulated in this memorandum” (p. 3) [22]. Research timeliness is vital to health professionals’ accuracy and efficacy, whether in treating or advocating for patients, addressing new health studies in the news, responding to public health emergencies, or keeping up with public health legislation and policy.

Also important are mechanisms for encouraging compliance with open access policies, including mandates such as the NIH policy. In 2012, it was reported that compliance with the NIH Public Access Policy had reached 82% [23]. At that point, PubMed Central held 2.4 million open access articles, with publishers, representing about 1,000 journals, voluntarily depositing more than 100,000 papers to the repository each year [3]. In November 2012 the NIH announced that it would start withholding grant money for noncompliance, leading to an apparent surge in new peer-reviewed articles deposited for public access. During May 2013 authors submitted more than 10,000 articles to PubMed Central, up from a monthly average of 5,100 in 2011–2012, approaching the agency’s “goal of getting everyone it funds to make their papers publicly available” [24].

This study’s finding that NGO participants’ view a greater proportion of open access articles, compared with that of physicians, appears to be due to the journals they consulted. For example, among the top twenty journals they viewed, *Environmental Health Perspectives* (published by the NIH’s National Institute for Environmental Health Sciences) did not appear on the physicians top-twenty list, while the NGOs viewed articles in the open access *British Medical Journal* (BMJ) and *PLOS ONE* more frequently than physicians.

## Future Research

Looking ahead, studies could investigate variations in open access publishing across health topics and professional fields, for a more fine-grained analysis of health research availability. The differential use of open access research by the two populations, and among the physicians based on year of graduation, in this study suggests the relevance of further investigations on needs, interests, and usage of research. In addition, research into the distinct and overlapping bodies of literature accessed by different professional communities via larger indices such as PubMed could suggest the challenges as well as possibilities for joint learning initiatives between the clinical medical and public health fields, as recommended by the Institute of Medicine [25] and other entities. Future studies could also examine the research uses, needs, and priorities of additional publics and health professionals, in relation to open access policies now in development across federal agencies. The mediating effects of different web portal designs, search tools, and training modules could be assessed. In addition, this study investigated the use of two research resources, UpToDate and PubMed, which, while billed as premier biomedical and life sciences information resources [12], are not the only research resources used by health professionals. Future studies could consider providing participants with a wider array of research resources to investigate participant needs. In terms of this study, we will also be following up with articles on participants’ research uses, practices and priorities in the course of their work.

## Limitations

This study must be considered within the context of its limitations. As this study provided no formal training or orientation to physicians or the majority of public health participants, searches performed with unfamiliar research resources may result in difficulty in finding relevant research. Both the study’s web portal and the research resources it featured (PubMed and UpToDate) may have presented learning curves and/or technical challenges to participants, interfering with research access and use. Though designed to be easy to use and access, it did require participants to have Internet access and keep the study informed of their current email addresses and access capabilities. Over the course of the year-long study, some participants and organizations experienced staff turnover that in turn impinged on study participation.

The web-oriented aspects of the study also present potential limitations. The extensive use of social media for recruitment of physicians creates possible bias toward attracting individuals already reliant on and comfortable using technology. Additionally, social media recruitment might under-recruit older participants or participants from regions reported to lack consistent access to technology [26]; however, researchers have reported that age may not predict social media use in the context of research literature [27]. Likewise, an Internet-based approach to identifying and recruiting public health organizations might over-recruit organizations with greater web visibility rather than any other consideration. Additionally, participants did not necessarily use the web portal exclusively for accessing research articles or UpToDate. Thus, they may have accessed the study’s portal only outside of their work environment. Such a limitation would lead to under-reporting potential interest in and need for research access.

## Conclusions

In light of the growing proportion of the literature that is publicly available, including via funding agency and publisher policies, these study findings underscore the potential utilization of open access to research by publics working to advance health through clinical medicine and the public health field, including the NGO sector. This study demonstrates that given a high level of research access and an awareness of that access, a sizeable portion of the physicians and public health NGO staff who participated in this study viewed full-text articles. Such article access facilitates fuller engagement with research developments across fields. Our findings also bear on the structure and implementation of the NIH Public Access Policy and similar initiatives. Based on the high proportion of articles accessed that had been published within the prior 12 months, it is clear that such embargoes represent a substantial potential impediment to research access. Together, these findings suggest the need for transformed models of public access to research, rather than inconsistent and occasional access. We trust that these results will encourage further tuning of policies, as well as the training and education of students in related professions in the utilization of the coming access. Given the demonstrable public value and public right to more fully benefit from this federally funded research, it is vital that funding agencies, legislators, researchers and publishers continue to explore ways of extending access to research that can serve the public good.

Better identification of policy stakeholders, coupled with tailored, pro-active leveraging of communications media, are both crucial to more effectively publicizing the NIH Public Access Policy. For example, studies of research dissemination by the public health field have called for “the need to change the way such information is disseminated so that it reaches different users in the most appropriate way,” including through “previous identification of the audience and taking advantage of all channels of communication to assure that the information is communicated and marketed—not merely disseminated—to those who need to know” [28]. Likewise, the NIH and other federal agencies should communicate with the wide array of health stakeholders—including non-governmental organizations, local health departments and other governmental organizations, physicians, and the general public—in ways that publicize and actively market new research resources, whether through listservs, social media or other communication channels. Improved communication of research resources such as the NIH Public Access Policy and future federal agency policies would help tap the expertise of a wider range of partners in the common project of leveraging research on behalf of public health. It would also help take health communication “upstream” to policy-makers, program managers and other public health decision-makers positioned to develop preventative health measures with wide social benefits.

## Supporting Information

S1 Screenshot Screenshot of portal page. (TIF)
