# Supplementary figures and images for: In an Age of Open Access to Research Policies: Physician and Public Health NGO Staff Research Use and Policy Awareness
Source: PLoS One. 2015 Jul 22;10(7):e0129708. doi: 10.1371/journal.pone.0129708 (PMC4511689; doi:10.1371/journal.pone.0129708)

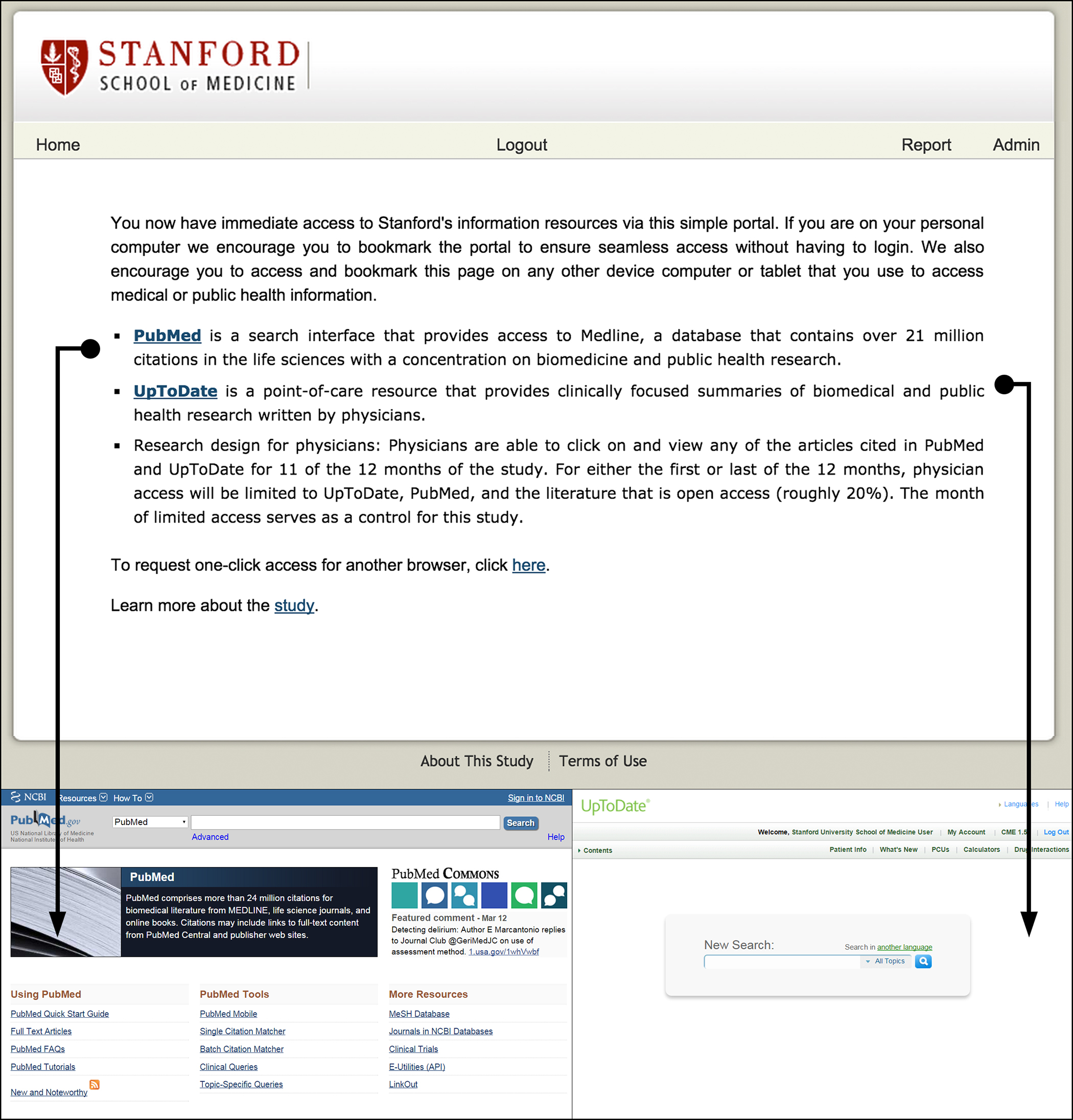

Supplement: S1 Screenshot — (TIF) [file pone.0129708.s001.tif]
